# Twist1 Contributes to the Maintenance of Some Biological Properties of Dermal Papilla Cells *in vitro* by Forming a Complex With Tcf4 and β-Catenin

**DOI:** 10.3389/fcell.2020.00824

**Published:** 2020-08-19

**Authors:** Nanlan Yu, Tianxing Hu, Haichao Yang, Lian Zhang, Qin Song, Fei Xiang, Xichuan Yang, Yuhong Li

**Affiliations:** ^1^Department of Dermatology, The First Affiliated Hospital of the Army Medical University, Chongqing, China; ^2^Department of Cell Biology, Army Medical University, Chongqing, China; ^3^State Key Laboratory of Trauma, Burns and Combined Injury, Southwest Hospital, Institute of Burn Research, Army Medical University, Chongqing, China

**Keywords:** Twist1, Tcf4, β-catenin, dermal papilla cells, hair follicle stem cells

## Abstract

**Objective:**

During hair follicle regeneration, hair follicle stem cells (HFSCs) are regulated by signals from dermal papilla cells (DPCs). Previously we found that Tcf4 could promote the proliferation of DPCs. In this study, we focused on whether and how the biological properties of Tcf4-induced DPCs were regulated by Twist1.

**Methods:**

Twist1 was overexpressed or knocked down in DPCs following different adenovirus or lentivirus infection. Phase-contrast microscopy was used to observe the agglutinative growth of DPCs. The CCK-8 assay was used to test the proliferation of DPCs. Western blot and qPCR experiments were used to determine the expression of HGF, IGF-1, VEGF, c-myc, survivin, and CyclinD1 in DPCs. ELISAs were used to test the growth factors secreted by DPCs. Conditional medium culture was used to detect the inductive ability of DPCs. Co-immunoprecipitation and immunofluorescence were used to test the binding of Twist1, Tcf4, and β-catenin in DPCs. Immunofluorescence was also used to test the expression of Twist1, Tcf4, and KRT15 in hair follicles.

**Results:**

Twist1 induced DPC agglutinative growth and proliferation. Twist1 upregulated the expression of downstream target genes downstream of Tcf4, c-myc, survivin, in Tcf4-induced DPCs, as well as the expression and secretion of growth factors HGF, IGF-1, VEGF, which had the ability to induce hair follicle growth. The conditional medium from Twist1-treated DPCs increased the expression of KRT40 and MSX2 in HaCaT cells. Twist1 and Tcf4 co-localized in DPCs both *in vitro* and *in vivo.* Anti-Twist1 precipitated Tcf4 and β-catenin.

**Conclusion:**

These results indicate that Tcf4 and Twist1 play a synergistic role in regulating the hair follicle induction ability of DPCs. Twist1 functions by forming a ternary complex with Tcf4 and β-catenin. Thus, we report new data that elucidate whether and how Twist1 regulates some biological properties of DPCs.

## Introduction

Hair loss is one of the most common dermatological diseases. Alopecia, represented by androgentic alopecia or alopecia areata, may last for a long time, or easily relapse. It causes great mental strain for patients (Williamson et al., 2001; Hunt and McHale, 2005). At the same time, the hair itself also has physiological functions such as temperature regulation or skin protection, as well as roles in information interaction and beauty. It has been reported that some patients with alopecia problems, such as baldness, can even experience devastating physical and mental disorders such as anxiety and depression (Van Der Donk et al., 1994; Williamson et al., 2001; Fabbrocini et al., 2013; Yu et al., 2016). At present, the treatment methods for hair loss include drug treatments to promote hair growth and hair transplantation, which require hair follicles from the patients themselves (Miao et al., 2014). The hair follicle is an appendage of the skin, the structure of which is composed of epidermis and dermis, further, the follicle experiences the periodic stages of growth, degeneration, and quiescence (Al-Nuaimi et al., 2010). Dermal papilla cells (DPCs) are mesenchymal cells located at the hair follicle bulb. DPCs not only play a regulatory role in the hair follicle cycle but also play a maintenance and induction role, which is characterized by agglutinative growth behavior *in vitro* and the ability to induce the formation of new hair follicles during the embryonic and postnatal periods (Driskell et al., 2011). DPCs promote the formation and development of hair follicles through the interaction of various signaling pathways. For example, the BMP signaling pathway and Wnt/β-catenin signaling pathway are considered to be key signaling pathways in the activation of hair follicle stem cells (HFSCs) and regeneration of hair follicles (Wu et al., 2019). BMP signaling has an inhibitory effect on the activation of HFSCs. TGF-β signaling from DPCs can inhibit the inhibitory effect of BMP signal in HFSCs (Oshimori and Fuchs, 2012; Amberg et al., 2016). When the Wnt signal is activated, the cytoplasmic signal molecule β-catenin cannot be phosphorylated and it becomes enriched in the cytoplasm before being transferred into the nucleus, where it forms a complex with Lef/Tcf. Once there, β-catenin plays a role in transcription activation, thus transcribing the molecules that activate HFSCs and hair follicle regeneration (Li et al., 2013; Bejaoui et al., 2019).

In our previous study, we found that Tcf4, a family member of Lef/Tcf encoded by the *Tcf7L2* gene, was upregulated in DPCs in the anagen (Xiong et al., 2014). Tcf4 and β-catenin form a complex in the nucleus, which leads to the transcriptional activation of genes downstream of the Wnt/β-catenin signaling pathway. In addition, Tcf4 was highly expressed in low passage number DPCs *in vitro*. However, the ability of hair follicle induction and the mode of agglutinative growth are often lost in the course of the passage of DPCs, as well as the expression of Tcf4, which shows that Tcf4 is closely related to the activation of HFSCs and hair follicle regeneration. Moreover, another previous study also found that mitotic arrest defective protein MAD2B, can bind with Tcf4 and reduce Tcf4-mediated Wnt/β-catenin signaling activity, thus inhibiting the proliferation of DPCs. However, it is interesting that the knockdown of MAD2B did not affect the secretion function of DPCs induced by Tcf4. In contrast, it further promoted the proliferation of Tcf4-induced DPCs (Yu et al., 2017). Based on these reports, we propose that the cytokine-mediated effects of MAD2B knockdown may be counteracted by other factors.

Twist1 is a transcription factor with a basic helix loop helix (bHLH). The Twist1 and Wnt/β-catenin signaling pathways play important roles in the development of osteosarcoma, in which Twist1 promotes the expression of specific cell surface receptors in osteosarcoma cells via activating Tcf4-mediated Wnt/β-catenin signaling pathway (Wu et al., 2014). Thus, we hypothesize that Twist1/Tcf4 interaction can promote the proliferation of Tcf4-induced DPCs. Through *in vitro* and *in vivo* experiments, we found that Twist1 may be an important factor that regulates the aging of DPCs, and can delay the disappearance of some biological properties of DPCs during passage. In addition, we found that Twist1 performed these activities by forming a complex with Tcf4 and β-catenin.

## Materials and Methods

### Cell Culture

This study was approved by the Ethics Committee of The First Affiliated Hospital of the Third Military Medical University (ethics approval No. ky201977). All experiments were carried out in accordance with the relevant guidelines and regulations. The patients/participants provided their written informed consent to participate in this study. The scalp samples containing hair follicles were collected in the Department of Dermatology of The First Affiliated Hospital of the Third Military Medical University from patients with a pigmented nevus and sebaceous nevus, whose size was 0.5 × 0.5 cm ∼ 0.5 ×1 cm. After collection, they were immediately stored in 4°C sterile saline, and primary cells were extracted by a two-step enzyme digestion. Primary DPCs were cultured with DMEM containing 10% FBS and 100 U/L penicillin-streptomycin in a 37°C, 5% CO_2_ incubator. The medium was changed every 3 days until the primary cells stopped growing, and then the cells were passaged and cultured at a ratio of 1:2. Primary human fibroblasts were obtained and cultured as previously described Chen et al. (2017), and were kindly provided by Chunmeng Shi in the Third Military Medical University.

### Virus Construction and Infection

An adenoviral vector mediating overexpression of Tcf4 was generated previously (Yu et al., 2017). The empty vector pAdeno-MCMV-3Flag-T2A-mCherry was used to construct an adenovirus-mediated Twist1 (NM_000474) overexpression vector. The construction and amplification of the Twist1 overexpression vector was performed by OBIO Technology Corp., Ltd. The empty vector pLKD-CMV-mCherry-2A-Puro-U6-shRNA was used to construct the lentiviral vector mediating siTwist1 knockdown. The construction and amplification of the siTwist1 vector was performed by OBIO Technology Corp., Ltd. The target sequence is: 5′-gcaagattcagaccctcaa-3′.

Before virus infection, DPCs were seeded into 6-well plates at a density of 3 × 10^5^ cells per well. When DPCs grew to ∼70%, virus-mediated GFP, Twist1, siTwist1, Tcf4, Tcf4 and Twist1, Tcf4, and siTwist1 were added into the culture medium. Cell morphology was monitored under a microscope.

### Cell Proliferation Assay

DPCs were seeded into 96-well plates at a density of 1 × 10^3^ cells per well. After overnight culture, cells were divided into 6 groups and were separately infected with viral vectors expressing GFP, Twist1, siTwist1, Tcf4, Tcf4 and Twist1, Tcf4, and siTwist1. Twenty-four hours later, a 10 μL cell counting kit-8 (CCK-8) solution (Beyotime, Shanghai, China) was added to the culture. After incubation in the dark for 1.5 h, the absorbance optical density (OD) value was measured at a wavelength of 450 nm.

### Western Blot Analysis

DPCs were seeded into 60 mm dishes at a density of 1 × 10^6^ cells per dish. Forty-eight hours after viral infection, the cells were washed twice with cold PBS and then lysed with RIPA buffer (Beyotime, Shanghai, China). Cell lysates were collected and the protein concentration was measured with a BCA protein assay (Beyotime, Shanghai, China). Subsequently, the protein was separated by SDS-PAGE and transferred to a PVDF membrane. The membrane was then blocked with 5% non-fat milk in TBS, and incubated overnight at 4°C with diluted primary antibodies. The primary antibodies were as follows: GAPDH, c-myc, survivin, CyclinD1, IGF-1, HGF, VEGF (R&D Systems, Minneapolis, MN, United States), Twist1 (Santa Cruz, CA, United States), KRT40 (Santa Cruz, CA, United States), MSX2 (Santa Cruz, CA, United States), KRT5 (Sangon, Shanghai, China), KRT15 (Sangon, Shanghai, China), and FGF7 (Boster, Wuhan, China). After washing, the membrane was incubated with HRP-labeled secondary antibodies. A Bio-Rad imaging system was used to collect and quantify images.

### Quantitative Polymerase Chain Reaction (qPCR)

Total RNA was extracted from DPCs with an RNA extraction kit (Takara, Dalian, China) and cDNA was synthesized by reverse transcription (Toyobo, Osaka, Japan). Quantitative polymerase chain reactions (qPCRs) were carried out with the following primers: 5′-ccgggatgctttacgttg-3′ (forward) and 5′-aattcaaaaagatgcagctta-3′ (reverse) for c-myc, 5′-ctttacgtggac tccagtct-3′ (forward) and 5′-acaagacctcaactgtggctcga-3′ (reverse) for survivin, 5′-agatgcagctttacgtggaagactcga-3′ (forward) and 5′-tacgtggaagactcgagtcttgtaa-3′ (reverse) for cyclin D1, 5′-cag cagtcttccaacccaat-3′ (forward) and 5′-cacgaactgaagagcatcca-3′ (reverse) for IGF-1, 5′-cagagggacaaaggaaaagaag-3′ (forward) and 5′-atgctattgaaggggaaccag-3′ (reverse) for HGF, 5′-gtcca acttctgggctgtct-3′ (forward) and 5′-ccctctcctcttccttctcttc-3′ (reverse) for VEGF.

### ELISA

The concentrations of IGF-1, HGF, and VEGF in the medium were determined by ELISA kits (Boster, Wuhan, China) according to the manufacturer’s instructions. The optical density (OD) was measured at 450 nm by a Varioskan Flash reader (Thermo Fisher Scientific, MA, United States).

### Conditional Medium Culture

DPCs were infected with viruses as mentioned before. At 24, 48, and 72 h after infection, the culture medium was changed. The medium at 48 and 72 h later was collected, mixed, and stored in a 4°C refrigerator. HaCaT cells were cultured in 1,640 culture medium with 10% FBS. At 24 and 48 h after seeding the cells, the culture medium was changed to conditional medium from the DPC culture. At 72 h after seeding, the total protein and total RNA of the HaCaT cells were extracted as mentioned before, and they were analyzed by Western blot or qPCR.

### Immunofluorescence

DPCs were cultured overnight in 35 mm glass dishes at a density of 1 × 10^5^ cells per dish. The next day, the DPCs were fixed with 4% paraformaldehyde. For paraffin-embedded scalps, the samples were sectioned into 5 μm sections and gradually hydrated. Then the sections were microwaved in citrate buffer (pH 6) for antigen retrieval and rinsed in PBS. Then, the DPCs or sections were treated with 0.1% Triton X-100, blocked with 5% BSA, and incubated overnight at 4°C with anti-KRT15 (Sangon, Shanghai, China), anti-Tcf4 (Proteintech, Shanghai, China), and anti-Twist (Santa Cruz, CA, United States). After washing, the DPCs or sections were incubated with Alexa Fluor 488-labeled donkey anti-rabbit and cy3-labeled goat anti-mouse secondary antibodies, then, they were counterstained with DAPI and observed under a fluorescence microscope.

### Co-immunoprecipitation (Co-IP)

Cultured DPCs were lysed with RIPA buffer (Beyotime, Shanghai, China), the protein concentration was measured with BCA, and a small part of the lysate was prepared as input. The rest of the lysate was incubated with immunoglobulin G (IgG; negative control), anti-Twist1 (Santa Cruz, CA, United States), or anti-Tcf4 (Proteintech, Shanghai, China) overnight. Then protein A/G agarose was added to couple to the antibody. Finally, the immunoprecipitate was eluted and analyzed by Western blot.

## Results

### Twist1 Promotes the Growth of DPCs

**FIGURE 1:**
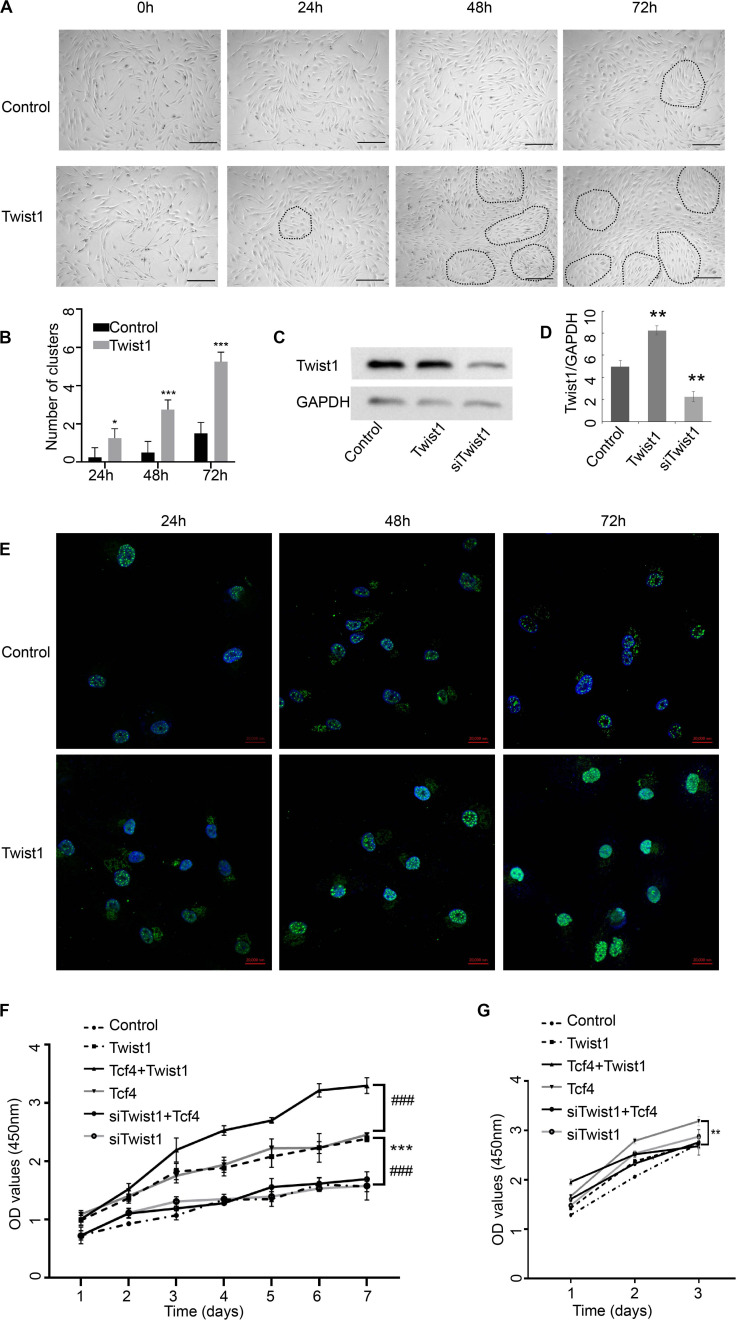
The impact of Twist1 on the growth of DPCs. **(A,B)** Changes in the morphology of Twist1 overexpressing DPCs. Dashed lines depict the DPC clusters. Scale bar = 50 μm. **(B)** Statistical analysis of **(A)**. **(C,D)** The overexpression and knockdown efficiency of Twist1 vectors. The expression of Twist1 was detected by Western blot. **(D)** Statistical analysis of **(C)**. The expression of Twist1 was normalized to that of GAPDH. **(E)** The expression of Twist1 detected by immunofluorescence in Twist1 overexpressed DPCs. DAPI was used to counterstain the nucleus. Scale bar = 20 μm **(F)** The growth curve of DPCs. **(G)** The growth curve of human fibroblast cells. Equal amounts of DPCs or human fibroblast cells were treated with virus vectors expressing GFP, Twist1, siTwist1, Tcf4, Tcf4, and Twist1, as well as Tcf4 and siTwist1. The proliferation of cells was detected by CCK-8 assay every day from 1 day after treatments. OD, optical density. *N* = 3. ^###^*P* < 0.001 when compared with the Tcf4-treated group. *P < 0.05 when compared with the control group. **P < 0.01 when compared with the control group. ***P< 0.001 when compared with the control group.

Agglutinative growth is one of the characteristics of intact DPCs. To determine whether Twist1 affects the agglutinative growth of DPCs, an adenovirus mediating overexpression of Twist1 was added to cultured DPCs. At 48 and 72 h after overexpression of Twist1, the agglutinative growth behavior of DPCs increased significantly. In contrast, the DPCs in the control group maintained aggregation behavior but did not have the characteristics of agglutinative growth (Figures 1A,B). However, the traditional aggregative behavior of DPCs has been demonstrated by self-aggregation of cultured DPC to form three-dimensional cell aggregates (Kiratipaiboon et al., 2015; Topouzi et al., 2017). We did not observe any three-dimensional cell aggregates in our two-dimensional culture system. The agglutinative growth of DPCs in this manuscript is defined as DPCs gather in clusters when growing. The DPC clusters were depicted out with dashed lines in Figure 1A. These data indicate that Twist1 plays a specific role in promoting the agglutinative growth of DPCs.

Adenovirus-mediated Twist1 expression or lentivirus-mediated siTwist1 expression effectively overexpressed or knocked down the expression level of Twist1, respectively (Figures 1C,D). Immunofluorescence assay demonstrated that the cells in the Twist1-overexpressing conditions maintain high Twist1 expression until 72 h after treatments (Figure 1E). Because our previous studies showed that Tcf4 can promote the proliferation of DPCs *in vitro*, we next tried to determine whether Twist1 played a role in the proliferation of DPCs induced by Tcf4. The proliferation rate of DPCs infected with adenovirus-mediated Tcf4 alone increased significantly compared with the control. Interestingly, the proliferation rate of DPCs infected with the virus expressing Tcf4 and siTwist1 was significantly lower than that of DPCs infected with the Tcf4 virus alone, which indicated that the knockdown of Twist1 could inhibit the proliferation of DPCs induced by Tcf4. The proliferation rate of the group co-infected with virus expressing Tcf4 and Twist1 was significantly higher than that of the other groups, which indicated that overexpression of Twist1 could further enhance Tcf4-induced DPC proliferation (Figure 1F). The above results imply that Twist1 may interact with Tcf4 in regulating DPC proliferation. We also tested the proliferation of human dermal fibroblast by CCK-8. The dermal fibroblast grew too fast, so we only tested it for 3 days. The effect of Twist1 on dermal fibroblast was different from its effect on DPCs (Figure 1G).

### Twist1 Promotes the Expression of Tcf4-Induced Target Genes in DPCs

**FIGURE 2:**
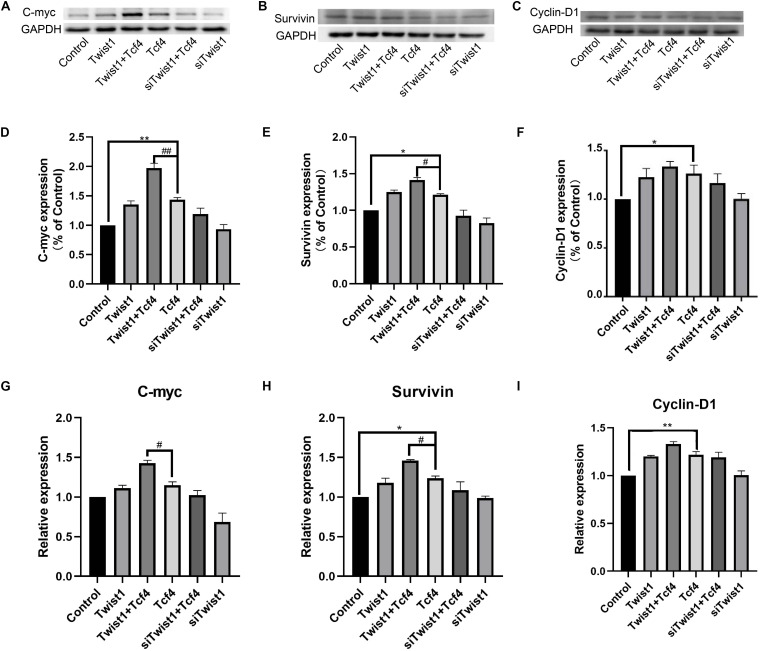
The expression of Tcf4 target genes in DPCs treated with Twist1, Tcf4, and siTwist1. **(A–C)** Western blot results of c-myc, survivin, and cyclinD1. **(D–F)** Quantitative analysis of **(A–C)**. The expression levels of target genes were standardized to that of GAPDH. **(G–I)** qPCR results of c-myc, survivin, and cyclinD1, the expression level of target genes was standardized to that of GAPDH. *N* = 3. ^#^*P* < 0.05, ^##^*P* < 0.01 when compared with the Tcf4-treated group. *P < 0.05, **P < 0.01 when compared with the control group.

To further research the functional interaction between Twist1 and Tcf4, the effect of Twist1 overexpression on the expression of target genes downstream of Tcf4 (including proto-oncogene c-myc, apoptotic protein survivin, cell cycle protein cyclinD1) was detected in Tcf4 overexpressing DPCs. Both overexpression of Tcf4 and Twist1 promoted the mRNA and protein expression of these downstream target genes in DPCs. Overexpression of Tcf4 and Twist1 together effectively promotes the mRNA and protein expression of c-myc and survivin. The overexpression of Twist1 did not significantly increase the expression of cyclinD1 when compared with Tcf4 overexpressing group. These results indicate that Twist1 is positively correlated with Tcf4-mediated activation of downstream target genes. However, knockdown of Twist1 in DPCs or Tcf4 overexpressing DPCs did not significantly change the expression of these target genes (Figure 2).

### Twist1 Promotes the Expression and Secretion of Tcf4-Induced Growth Factors in DPCs

**FIGURE 3:**
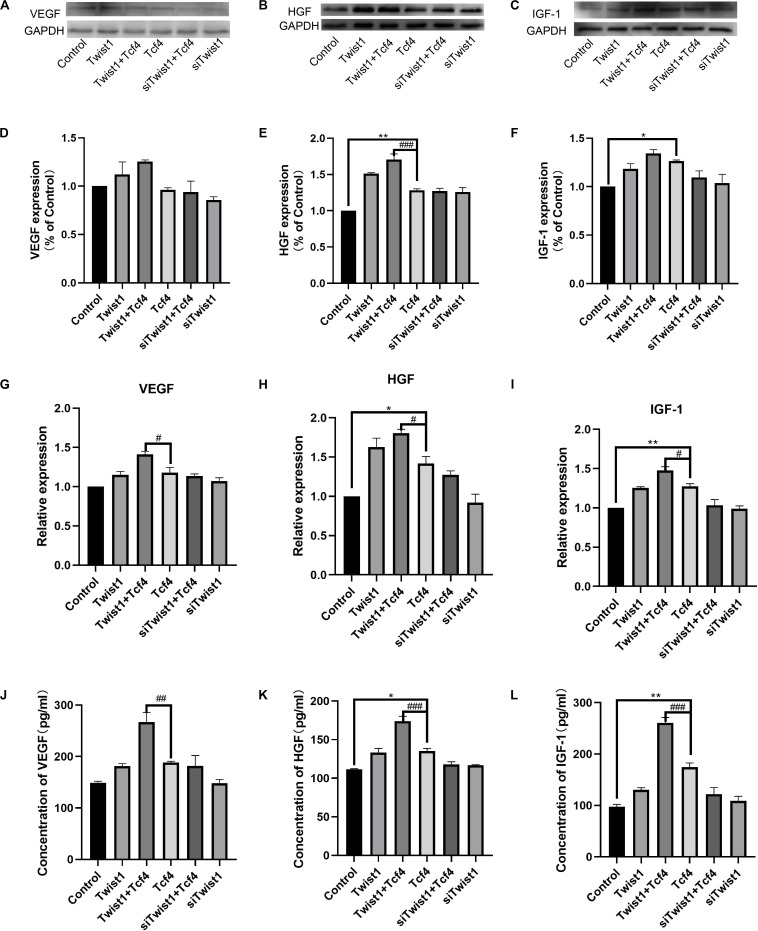
The expression and secretion of hair follicle inductive molecules in DPCs. **(A–C)** Western blot results of VEGF, HGF, and IGF-1. **(D–F)** Quantitative analysis of **(A–C)**. The expression levels of target genes were standardized to that of GAPDH. **(G–I)** qPCR result of VEGF, HGF, and IGF-1. The expression level of target genes was standardized to that of GAPDH. **(J–L)** Expression of VEGF, HGF, and IGF-1 detected by ELISA in the culture supernatant of DPCs. *N* = 3. ^#^*P* < 0.05, ^##^*P* < 0.01 when compared with the Tcf4-treated group, ^###^*P* < 0.001 when compared with the Tcf-treated group. *P< 0.05, **P < 0.01 when compared with the control group.

DPCs usually regulate hair follicle regeneration by secreting growth factors. To investigate the effect of Twist1 on hair follicle regeneration, the expression and release of hair growth-related growth factors (VEGF, HGF, and IGF-1) were detected after Twist1 was overexpressed in DPCs. Consistent with the role of Tcf4 in DPC proliferation, both overexpression of Tcf4 and Twist1 promoted the mRNA and protein expression of these growth factors in DPCs. Overexpression of Tcf4 and Twist1 together promoted the mRNA and protein expression of these growth factors more effectively than either factor by itself. On the other hand, knockdown of Twist1 in Tcf4-treated DPCs had a lower expression level of these growth factors than that of control cells. However, compared with the control, siTwist1 alone did not significantly change the expression of these growth factors (Figures 3A–I). The trends of the expression of these growth factors in the supernatant were the same as they were in DPCs (Figures 3J–L). These results indicate that Twist1 is positively related to the production and secretion of growth factors regulated by Tcf4.

### Twist1 Maintains Some Biological Properties of DPCs

**FIGURE 4:**
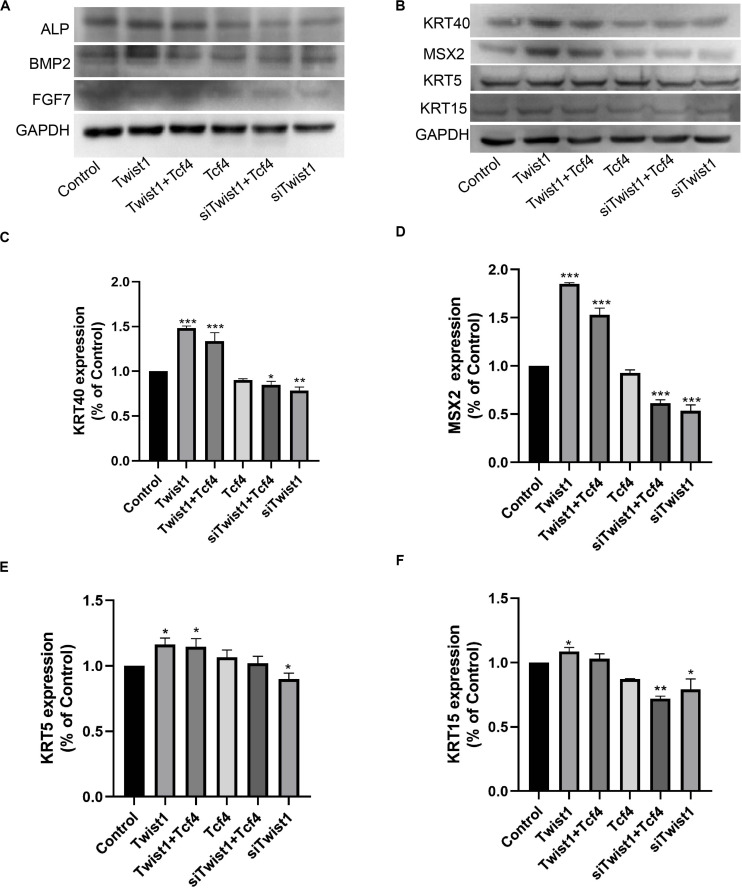
The inductive ability of DPCs. **(A)** The intact DPC markers ALP, BMP2, and FGF7 were detected by Western blot. **(B–F)** HaCaT cells were cultured with conditioned medium from DPCs treated with virus expressing Tcf4, Twist1, and siTwist1. Differentiation markers KRT40, MSX2, KRT5, and KRT15 were detected by Western blot. **(C–F)** Quantitative analysis of **(B)**. The expression levels of target genes were standardized to that of GAPDH. *P < 0.05 when compared with the control group. **P < 0.01 when compared with the control group. ***P < 0.001 when compared with the control group.

DPCs were treated with adenoviral vectors expressing Tcf4 and Twist1 or lentiviral vector expressing siTwist1 together or separately. After treatments, the DPCs still maintained expression of intact DPC markers (Figure 4A). HaCaT cells are from the basal layer of the skin, and can be induced to differentiate into cells resembling various types of skin cells. The conditioned medium from Twist1-treated DPCs induced HaCaT cells to express higher levels of KRT40, MSX2, KRT5, and KRT15 (Figures 4B–F). These results suggest that Twist1 can maintain the ability of DPCs to induce the differentiation of epidermal cells *in vitro*.

### Twist1 Physically Interacts With Tcf4 in DPCs

**FIGURE 5:**
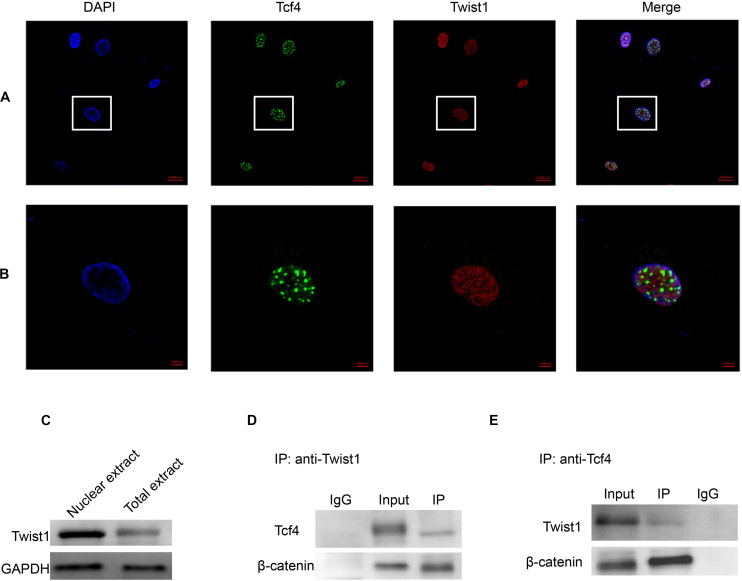
The interaction of Tcf4 and Twist1 in DPCs. **(A,B)** The expression of Tcf4 and Twist1 were detected by immunofluorescence in cultured DPCs. **(A)** Scale bar = 20 μm. **(B)** Scale bar = 5 μm. **(C)** The expression of Twist1 was determined by Western blot in nuclear extract and total extract from cultured DPCs. **(D)** The expression of Tcf4 and β-catenin in the anti-Twist1 precipitated complex in cultured DPCs. **(E)** The expression of Twist1 and β-catenin in the anti-Tcf4 precipitated complex in cultured DPCs.

The above data show that there is a functional interaction between Twist1 and Tcf4. Therefore, we next attempted to detect whether there was a physical interaction between Twist1 and Tcf4. Immunofluorescence showed that both Twist1 and Tcf4 were mainly distributed in the nucleus of DPCs. Interestingly, Tcf4 co-localized with a subset of Twist1 in the nucleus of DPCs (Figures 5A,B). Western blot analysis of total protein and nuclear extracts also showed that Twist1 was mainly expressed in the nucleus of DPCs (Figure 5C). In co-immunoprecipitation experiments, both Tcf4 and β-catenin were found to be precipitated by anti-Twist1, and both Twist1 and β-catenin were precipitated by anti-Tcf4 (Figures 5D,E).

**FIGURE 6:**
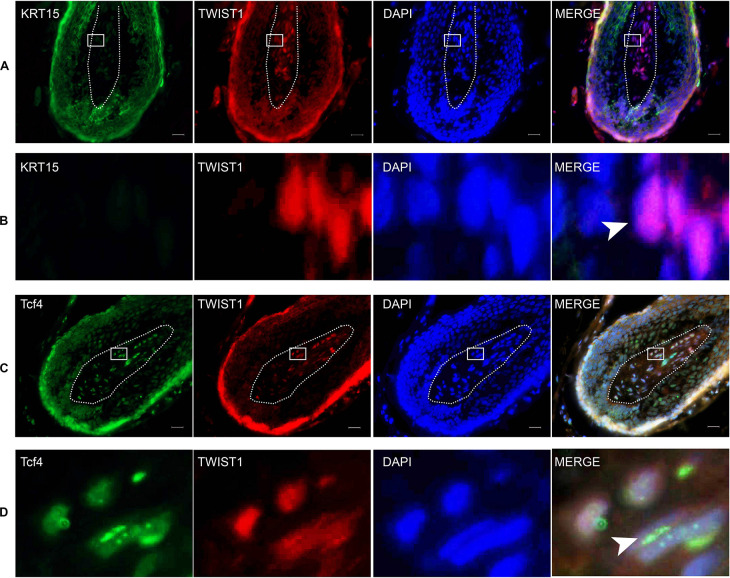
The expression of KRT15, Twist1, and Tcf4 in the scalp. The right panel is a merged image of the left three panels. Dashed lines depict the structure of the dermal papilla. **(B)** The enlarged picture of the framed area in **(A)**. Arrowhead denotes the positive expression of Twist1 in the nucleus. **(D)** Is the enlarged picture of the framed area in **(C)**. Arrowhead denotes the co-expression of Tcf4 and Twist1 in the nucleus. Scale bar = 20 μm.

To investigate the role of Twist1 in HFSCs and hair follicle regeneration, the expression of Twist1, Tcf4, and KRT15 was determined by immunofluorescent double labeling. In the hair follicle, KRT15 and Twist1 did not exhibit any kind of co-localization (Figures 6A,B). Whereas Tcf4 was colocalized with a subset of Twist1 in the nucleus of some Twist1 expressing DPCs *in vivo* (Figures 6C,D). These results demonstrate that Twist1 may physically interact with Tcf4 and β-catenin in DPCs.

## Discussion

Tcf4 is encoded by the *Tcf7L2* gene, and it is an important member of the Lef/Tcf protein family. In the process of tissue development and differentiation, Tcf4 responds to the β-catenin signal and initiates the downstream Wnt signaling pathway, thus promoting cell proliferation and differentiation (Cadigan, 2012). Disruption of Tcf4 or β-catenin can block the Wnt signaling pathway and lead to complete loss of cell proliferation (Ma et al., 2020). Our previous studies have shown that Tcf4 was highly expressed in growing hair follicles and in DPCs cultured *in vitro* when its morphological features were still in the agglutinative growth mode (Xiong et al., 2014). The Wnt signaling pathway mediated by the Tcf4/β-catenin complex plays an important role in hair follicle morphogenesis. However, it is not clear how Tcf4/β-catenin participates in the process of hair follicle induction and growth. In this study, we found that Twist1 could promote the proliferation of DPCs (Figure 1), and the overexpression of Twist1 could promote the expression of downstream target genes of Tcf4 and the secretion and release of growth factors in DPCs (Figures 2, 3). The function of Twist1 was consistent with that in a previous report (Shen et al., 2019). Therefore, Twist1 may be a positive regulatory partner of Tcf4. However, the knockdown of Twist1 did not affect the target gene expression, growth factors secretion or release of DPCs. One possible explanation for this difference is that the effects of downstream gene activation and transcription may be compensated for or replaced by other molecules.

DPCs located at the bottom of hair follicles can induce hair follicle regeneration and control the periodic growth of hair follicles. These characteristics of DPCs have been used to develop treatments for alopecia, including hair follicle autografts. It has been proven that DPCs cultured *in vitro* have the ability to induce hair growth only when they have the characteristics of agglutinative growth. At the same time, this agglutinative growth mode gradually disappears with an increase in the number of passages (Lei et al., 2017). Higgins et al. found that the transcription profiles of intact dermal papilla can be partially restored by culturing DPCs in 3D spheroid cultures (Higgins et al., 2013). Due to the decreased expression level of Tcf4 in DPCs during passages, there may be a correlation between Tcf4 expression and the agglutinative growth mode of DPCs. In this study, we first proved that Twist1 could promote the agglutinative growth behavior of DPCs *in vitro*, which shows that Tcf4 and Twist1 have a similar effect on the agglutinative growth behavior of DPCs. It is well known that downstream target genes of Tcf4 in Wnt signaling, c-myc, survivin and cyclinD1, are involved in cell growth and cell cycle regulation, and growth factors HGF, VEGF, and IGF-1 promote hair growth. C-myc was required to maintain the stem cell-like phenotypes of DPCs (Muchkaeva et al., 2014), which was a critical biological process that kept the high rate of hair growth (Kiratipaiboon et al., 2015). The expression level of survivin in DPCs is related to hair growth (Zhang et al., 2016). CyclinD1 is one of the main factors that regulates DPCs’ entry into the S phase (Kang et al., 2017). Our data demonstrated that Twist1 and Tcf4 co-overexpression increased the expression of c-myc and survivin, but did not increase the expression of cyclin D1 distinguishably, when compared with Tcf4 overexpressing group. Maybe cyclin D1 is very sensitive to Tcf4 and Twist1 in this case, so that the overexpression of Twist1 or Tcf4 alone had already increased the expression of cyclin D1 to the highest level of the cell needed. HGF was first found to be a polypeptide factor that promotes cell mitosis in plasma and platelets and has been reported to activate hair follicle morphogenesis during the growth period (Fujie et al., 2001). IGF-1 is a structural homolog of insulin, which is expressed in mesenchymal cells of the hair bulb and dermis. It has a significant impact on the hair follicle cycle and plays an active role in regulating hair follicle development (Ben Amitai et al., 2006). VEGF is a vascular endothelial factor that can promote hair growth by inducing capillary formation around hair follicles (Zhang et al., 2019). Consistent with these results, Tcf4 and Twist1 increased the mRNA and protein levels of c-myc, survivin, and cyclin-D1 and the secretion of HGF, VEGF, and IGF-1 (Figures 2, 3). In addition, conditioned medium from Twist1-treated DPCs induced HaCaT cells to express differential markers (Figure 4). These effects were enhanced when Tcf4 and Twist1 were overexpressed together, indicating that Twist1 had a positive effect on the maintenance of some biological properties of DPCs.

Twist1 is an autosomal bHLH transcription factor. Its structure includes an N-terminal, a C-terminal, and a highly conserved bHLH domain. The domain with DNA binding sites is the main domain required for Twist1 to function as a transcription factor. Epithelial-mesenchymal transition (EMT) is very important for the development of metastatic diseases. Twist1 is considered to be an inducer of EMT and a basic regulator of some metastatic diseases (Shen et al., 2019). It has been reported that skin formed by the interaction of epithelial and mesenchymal components. The hair follicle has become one of the main models to research this special regeneration structure. Hair follicle epithelium and HFSCs respond to signals from DPCs (Lei et al., 2017). Because Twist1 is a positive regulator of TCF4, Twist1 may play a key role in regulating the Wnt signaling pathway as it relates to DPC growth and hair follicle induction. However, from our data, we found that only a subset of Twist1 expressing cells expressed Tcf4, and only a sunset of Twist1 expressing foci also expressed Tcf4 in the cell nucleus. All Tcf4 expressing cells or foci expressed Twist1. These expression patterns suggested that Twist1 might have other functions in the cell.

## Conclusion

In conclusion, we reported that Twist1 promoted the agglutinative growth of DPCs, promoted the proliferation of DPCs induced by Tcf4 and promoted the expression of target genes of Tcf4 and the secretion of growth factors that regulate HFSC activation and hair follicle regeneration. We also reported that Twist1 functioned by forming a ternary complex with Tcf4 and β-catenin. Thus, we report new data that elucidate whether and how Twist1 regulates some biological properties of DPCs *in vitro*. Targeting the Twist1/Tcf4 complex is a potential treatment strategy for hair loss.

## Data Availability Statement

All datasets generated for this study are included in the article/supplementary material.

## Ethics Statement

This study was approved by the Ethics Committee of The First Affiliated Hospital of the Third Military Medical University (ethics approval no. ky201977). The patients/participants provided their written informed consent to participate in this study.

## Author Contributions

XY and YL designed the conceptual idea for this study. NY and TH performed most of the experiments. HY and LZ performed some of the experiments. QS and FX analyzed the data. NY and YL wrote the manuscript. All authors approved the submission of this manuscript in its final form.

## Conflict of Interest

The authors declare that the research was conducted in the absence of any commercial or financial relationships that could be construed as a potential conflict of interest.
